# Engineering of the LukS-PV and LukF-PV subunits of *Staphylococcus aureus* Panton-Valentine leukocidin for Diagnostic and Therapeutic Applications

**DOI:** 10.1186/1472-6750-13-103

**Published:** 2013-11-19

**Authors:** Charles Emeka Okolie, Alan Cockayne, Christopher Penfold, Richard James

**Affiliations:** 1 School of Molecular Medical Sciences, Centre for Biomolecular Sciences, University of Nottingham, University Park, Nottingham NG7 2UH, UK; 2 Department of Biological Sciences, College of Science and Engineering, Landmark University, Omu-Aran, Kwara State, Nigeria

**Keywords:** *Staphylococcus aureus*, Panton-Valentine leukocidin, Leukocytolytic exotoxin, Chromatography, Mass spectrometry, Immuno-therapy

## Abstract

**Background:**

*Staphylococcus aureus* produces several toxins, including Panton-Valentine leukocidin (PVL). The involvement of PVL in primary skin infections, necrotizing pneumonia, musculoskeletal disorders, brain abscess, and other diseases, some of which are life-threatening, has been reported. Following expert opinion, we aimed to provide the tools for establishment of sequence-based diagnostics and therapeutics for those conditions. We engineered the synergistic S and F (LukS-PV and LukF-PV respectively) pro-toxin subunits from *Staphylococcus aureus* USA400 into separate expression *E. coli BL21*(*DE3*)-*pLysS* hosts.

**Results:**

Following Nickel affinity chromatography (NAC), the F subunit came out without bands of impurity. The S sub-unit did not come off very pure after NAC thus necessitating further purification by size exclusion and ion-exchange chromatography. The purification plots showed that the BioLogic-LP and AKTA systems are reliable for following the progress of the chromatographic purification in real-time. Computer predicted Mw for the 6His-LukF-PV and 6His-LukS-PV were 35645.41 Da and 33530.04 Da respectively, while the mass spectrometry results were 35643.57 Da and 33528.34 Da respectively.

**Conclusion:**

The BioLogic-LP and AKTA systems are commendable for reliability and user-friendliness. As a recent work elsewhere also reported that a second round of chromatography was necessary to purify the S subunit after the first attempt, we speculate that the S subunit might contain yet unidentified motif(s) requiring further treatment. The purified S and F sub-units of PVL were supplied to the Nottingham Cancer Immunotherapy group who used them to establish sequence-based monoclonal antibodies for diagnostic and therapeutic uses targeting PVL.

## Background

Among two-component synergo-hymenotrpoic toxins associated with the genus Staphylococcus, leukocidin (Luk), gamma-hemolysin (Hlg), and Panton-Valentine leukocidin (PVL) have been well documented [1-3]. The synergo-hymenotrpoic toxins are encoded by distinct genetic loci [3]. *Staphylococcus aureus* PVL is encoded by two co-transcribed genes, *lukS*-*PV* and *lukF*-*PV*[4] which reside in the genomes of several bacteriophages associated with various different strains of *S. aureus*[3,5,6]. The active form of PVL requires the assembly of the two corresponding polypeptides, respectively LukS-PV and LukF-PV. The prophage harbouring PVL-encoding genes in *S. aureus* strain USA400 (also known as *S. aureus* strain MW2) is *phiSa2MW*[7,8].

Originally described in association with pus formation, carbuncles and furuncles [9], PVL did not receive much attention in scientific literature because the pathogenicity of *S. aureus* was formerly perceived to be restricted to secondary infections [10]. PVL-positive *S. aureus* (PPSA) was first associated with primary skin infections in France [11]. Following a report associating PVL with primary skin infections and pneumonia [12], reports of the prevalence of primary human diseases associated with PPSA, including highly contagious therapy-refractory skin infections and life-threatening haemoptysis, have been on the increase all over the world [13,14].

In Germany, Jung and colleagues reported the case of a 23-year-old apparently healthy female patient without any typical predisposing findings who developed severe sepsis with necrotizing pneumonia and multiple abscesses following incision of a Bartholin's abscess [15]. Methicillin-sensitive *S. aureus* harbouring PVL genes were cultured from the abscess fluid, multiple blood cultures and a postoperative wound swab. Aggressive antibiotic therapy with flucloxacillin, rifampicin and clindamycin, drainage and intensive supportive care lead finally to recovery. In their concluding recommendation, PPSA strains, should be considered when a young, immunocompetent person develops a fulminant necrotizing pneumonia as minor infections, such as Bartholin's abscess, can precede this life-threating syndrome. That was probably the first use of the word “SYNDROME” in the description of PVL-associated clinical conditions. Other signs and symptoms of the PVL-syndrome include multiple organ failure preceding acute respiratory distress syndrome (ARDS) such that the victim deteriorate or die rapidly (average: <72 hours, reports of <24 hours have been documented) from overwhelming toxicity [15-18]. Other reports from Germany [13,16], Denmark [17,18], France [19], Trinidad & Tobago [20] underline the existence of a "*Staphylococcus aureus* PVL-syndrome" independent of susceptibility to meticillin.

Furthermore, the body organs/systems involved including the skin, brain, respiratory and musculo-skeletal organs [21-23], points to PVL as a new worldwide public health threat. In Spain, a 34-year-old man was admitted for right parietal brain abscess and thickened dura mater in close proximity to a lytic bone lesion. The abscess culture yielded PVL-positive community-acquired meticillin susceptible *S. aureus* (PPCAMSSA). The patient survived after surgery and antibiotic treatment. This was the first reported case of a brain abscess due to PPSA [24]. In Egypt, a 50-year-old man was diagnosed with brain abscess associated with PVL-positive community acquired-MRSA (PPCAMRSA) in April 2007; following two intensive care admissions, his death was reported at the end of July 2007 [25]. Survivors of PVL-syndrome often go through rigorous intensive care [15,23,25,26]. Other than the 50-year old Egyptian man, the victims of PVL syndrome are often young healthy persons, including school children (mean age = 20) [14,15,17].

Molecular characterization have shown that PVL-positive *S. aureus* (PPSA) belong to different genotypes [27] and could be acquired from the community [16,25,27] as well as from the hospital [21]. Conventionally optimal medication and management is not effectual and no therapeutic preparation is particularly successful [26,28] as gold standard treatment is lacking [29,30]. Treatment is further complicated because the outcomes of PVL-associated conditions are independent of *S. aureus* meticillin susceptibility, while expert opinion signals bleakness “Until the toxins and inflammatory intermediaries responsible for the necrosis can be neutralised earlier,” [29,30].

The use of immune bodies for the treatment of toxic *S. aureus* infection has been reported, including septicaemia which was successfully treated with immune blood [31]. Therefore there is hope for immunoglobulin therapy. Human polyclonal immunoglobulin containing anti-PVL antibodies (Tégélin^R^) was proposed as a potential treatment for PVL necrotizing pneumonia [32]; but, not much was reported subsequently of Tegelin. Thus the need for rapid and specific therapy against PVL syndrome, and the attendant rapidity of victim deterioration, remains to be met.

To provide the tools for the establishment of therapeutic monoclonal antibodies as indicated by expert opinion, we cloned the two genes (*lukS*-*PV* and *lukF*-*PV*) coding for LukS-PV and LukF-PV respectively from *S. aureus* USA400 into two separate expression *E. coli BL21*(*DE3*)-*pLysS* host systems from which we sub-cloned each of them into two separate expression *E. coli* host systems. Following expression of the constructs, which we confirmed using Coomassie stained sodium dodecyl sulphate-polyacrylamide gel electrophoresis (SDS-PAGE) gels, the pro-toxins were purified by chromatography. The molecular masses of the purified peptides were determined by electrospray ionization–time-of-flight (ESI–TOF) mass spectrometry.

## Methods

### Gene cloning and expression protocols and reagents

Except otherwise stated, gene cloning and protein expression protocols were based on standard molecular cloning textbook [33] with few modifications.

### Bacterial strains and plasmids used for Recombination

The bacterial strains and plasmids used for recombination are described in Table 1.

**Table 1:** Bacterial isolates and plasmids used for genetic engineering

**Identification**	**Genetic property**	**Source**
*S. aureus MW2* (USA400)	PVL-positive CA-MRSA^a^	Network on antimicrobial resistance in *Staphylococcus* aureus (NARSA)
pGEM^®^-T Easy Vector	Intermediate plasmid vector	Lab stock maintained by Dr. Philip Bardelang [34]
*Escherichia coli DH-5α*	Intermediate *E. coli* host	
pET-21d(+)	Protein expression plasmid vector	
*E. coli BL21(DE3)pLysS*	Protein expression *E. coli* host	

a CA-MRSA is short for Community acquired meticillin resistant *Staphylococcus aureus.*

### Cultivation of bacteria and extraction of bacterial genomic DNA

Due to the unascertained risk level of *S. aureus* strain USA400 (also called MW2), isolation and DNA extraction were performed in biosafety level 3 (BSL3) containment laboratory designated for work with pathogens suspected to be potentially hazardous. It is required by CDC and NIH guidelines that when uncertain of the risk level, a presumably higher protection level should be applied [35]. Sterile disposable loop was used to touch frozen culture and plated out on brain heart infusion (BHI) agar. Following overnight incubation (18-28 hours) at 37°C, genomic DNA was extracted from the grown cultures by suspending four discrete colonies in 1.0 ml of molecular grade nuclease-free water (Sigma, UK) in a 1500 μL PCR grade eppendorf tube (Eppendorf, Germany). The tube was heated (98°C, 10 minutes) and then centrifuged (13000 g, 30 seconds) in an Eppendorf benchtop microcentrifuge (Eppendorf, Germany). The supernatant was taken out of the level 3 suite and used for PCR in the general (BSL2) laboratory.

### PCR amplification of recombinant *lukS*-*PV* and *lukF*-*PV* from *S. aureus* USA400

Suitable primer sequences were identified within the *lukS-PV* and *lukF-PV* genes of the PVL coding region of *S. aureus* strain MW2 (GenBank BA000033). The 5´ primers were chosen within the coding sequence of each gene, omitting the region predicted to encode the signal peptide as published for PVL [4] and verified online (http://www.cbs.dtu.dk/services/SignalP/). On account of previous success in our laboratory [34], we replaced the signal sequence of each pro-toxin subunit with *NcoI* sequence, which inserts a methionine start codon; we also replaced the stop codons with *XhoI* sequence, which enables the insertion of 6His-tag to allow for interactions between poly-histidine fusion tags and immobilized metal ions for protein purification. The 3' primers encompassed the stop codon of each gene. *NcoI* site (C/CATGG) was engineered into the primer sequence for the 5' primers (*rlukS-PV-1* and *rlukF-PV-1*) and *Xho1* site (C/TCGAG) was engineered into the primer sequence for the 3´ primers (*rlukS-PV-2 and rlukF-PV-2*). We used *Pwo* DNA polymerase for PCR high yield and fidelity.

### Separate cloning of DNA fragments into intermediate *E. coli* host system

The two recombinant DNA fragments amplified from *S. aureus* MW2 were separately TA ligated with intermediate vector pGEM®-T Easy (Promega, Madison, USA). Since the DNA fragments were blunt-ended PCR products of high fidelity *Pwo* polymerase, *Taq* DNA polymerase was used for 3’-A tailing to allow for ligation with the complementary T projecting from the pGEM®-T Easy plasmid. To allow for 3´-A overhangs necessary for the TA cloning principle of the pGEM®-T Easy vector, 3’-A tailing by *Taq* DNA polymerase is often performed in our laboratory. Briefly, gel-purified PCR product (4 μL) was transferred into a 200 μL PCR tube maintained on ice and containing thermopol buffer (2 μL), 1.0 μM of d’ATP and 5 units of *Taq* polymerase (Roche, Germany) all in a total volume of 10 μL. The mixture was vortexed for thorough mixing and incubated on the cycler (72°C, 10 minutes) to enable 3´-A tail. The resulting 3´-A tailed reaction was placed on ice and used immediately for ligation reaction with pGEM®-T Easy plasmid. Aliquot (2 μL) of the ligation was transformed into *Escherichia coli* 5α and subsequently plated out on Luria-Bertany (LB) agar containing ampicillin (100 μg/mL) and X-gal (40 μg/mL) for easy distinguishing of recombinant (white) colonies from non-recombinant (blue) transformant colonies with further confirmation by PCR amplification.

### Sub-cloning of separate insert DNA fragments from intermediate *E. coli* host system into expression *E. coli* host system

One white colony of the transformant from recombinant *E. coli* clones was subcultured in LB broth (Difco Laboratories, Detroit, MI) to obtain plasmid miniprep which was purified using Wizard® SV system (Promega, USA). Each separate fragment of insert DNA (S or F subunit) was released from the intermediate plasmid DNA (pGEM®-T Easy plasmid vector) by double-digest of the miniprep. The double-digest reaction (40 μL) contained 15 uL of the intermediate plasmid miniprep, 4 μL of X10 multicore buffer (promega, UK), 4 μL of X10 Bovine Serum Albumin (Promega, UK) and 0.5 μL of *NcoI* (NEB, UK) and 0.5 μL of *XhoI* (NEB, UK). The double-digests were incubated for 4 hours at 37°C. The expression plasmid vector pET-21d (+) (Novagen, USA), was gated and linearized by the same double-digest. Following digests, the restriction digest enzymes were inactivated by heating at 65°C for 15 minutes according to manufacturer’s instructions. Shrimp alkaline phosphatise (SAP), according to the manufacturer’s instruction (Promega, UK), was used to prevent the linearized vector plasmid from recircularizing. Briefly, 20 μL from the 40 μL doubly-digested pET-21d(+) was transferred to a fresh sterile 500 uL Eppendorf tube and made up to 25 μL of dephosphorylation mix by adding 3.0 μL of SAP 10X reaction buffer (equivalent to 50 mM Tris–HCl, pH 9.0 at 37°C, and 10 mM MgCl_2_) and then 1.0 μL equivalent to 1.0U of SAP. Dephosphorylation was incubated at 37°C for 1 hour. SAP was inactivated by heating at 65°C for 15 minutes. The inserts were then ligated separately with the expression plasmid vector using T4 DNA ligase (NEB, UK). Each ligation reaction for the expression contained 2 μL of the digested insert, 2 μL of pET cut with the same RDEs, 1.0 μL of X5 DNA buffer, 5.0 μL of X2 ligase buffer and 1.0 μL of T4 DNA ligase. The ligation reaction was incubated on the Eppendorf cycler at 22.5°C for 5 minutes. The contents of the ligation reaction tube were concentrated to the bottom of the tube by centrifugation (30,000RPM, 1 minute). A volume of 4 uL of the ligation reaction was used to transform one aliquot of expression *E. coli BL21*(*DE3*)-*pLysS* cells (Invitrogen, Paisley, UK). The transformation reaction was maintained on ice for 30 minutes and heat-shock at 42°C for 45 seconds and returned to ice for 2 minutes. Pre-warmed LB broth (400 μL at 37°C) was added to the transformation reaction which was then maintained at 37°C for 90 minutes in the shaker incubator. Aliquots (20 μL) were then plated out on LB agar supplemented with ampicillin (100 mg/L) and incubated overnight (24 hours). Growth on ampicillin was used as evidence of successful transformation. Transformant *E. coli* clones were further confirmed by PCR. The resulting plasmids were transformed into the expression *E. coli BL21*(*DE3*)*pLysS* and plated out on LB agar containing ampicillin (100 μg/mL). Growth on LB agar containing ampicillin (100 μg/mL) was was the phenotypic evidence of successful transformation.

### Structural confirmation of fusion DNA and translated peptide sequences

Transformant expression *E. coli* clones were further confirmed by PCR amplification and sequencing of the appropriate DNA insert (*rlukS*-*PV* and *rlukF*-*PV*) from the respective expression *E. coli* hosts using the same primers engineered with *NcoI* and *XhoI* sequences with which the template DNA was amplified from *S. aureus* MW2 at the beginning of the cloning experiment. To confirm the structural integrity of each reading frame, including the insertion of the N-terminal T7-Tag® sequence and the C-terminal 6His-Tag® sequence, we sent the purified plasmids to Cambridge Geneservice (http://www.geneservice.co.uk/home/) for sequencing as we did not have the necessary sequencing primers in Nottingham at the time. We then used the sequence translation tool at the European Bioinformatics Institute (transeq) to translate the nucleotide sequences from Geneservice into peptide sequences.

### Expression of recombinant proteins

Transformant *E. coli* clone (1 discrete colony), confirmed by PCR and sequencing, was picked into 200 mL of 2x YT broth in a 2 Litre flask and shaker-incubated at 35°C for 5 hours to mid-log phase (OD_600nM =_ 0.7). Uninduced control (1.0 mL) was removed for SDS-PAGE before adding isopropyl--D-thiogalactopyranoside (IPTG) stock of 839 mM (Fisher, UK) to achieve a 1.0 mM final concentration. The induction was incubated at 30°C with shaking at 200 rpm for 4 hours in a Model G25 controlled environment incubator shaker (New Brunswick Scientific, USA). An aliquot (10 μl) diluted in equal volume of double strength SDS loading buffer was tested on SDS-PAGE to confirm induction using prestained protein marker as a guide of protein size. Protein expression *E. coli* cells were spun down (13,000RPM, 1 minute) using Eppendorf bench top centrifuge. After discarding the supernatant, the cell pellet was dissolved in 100 uL of 2x SDS-PAGE loading buffer and heated on the cycler for complete lysis (98°C, 3 minutes) followed by another spin at 13,000RPM for 2 minutes. Using pre-stained protein marker to ascertain protein size, an aliquot of the protein rich supernatant (10 μL) was loaded onto a 10% SDS resolving gel through a 6% stacking gel. The gel was run first at 150 V for 15 minutes settling the samples at the interphase between the stacking gel and the resolving gel and then at 175 V for further 30 minutes. The gel was stained with Coomassie stain for 2 hours and then transferred to fast decolorizer for 30 minutes and finally transferred to and left overnight in slow destain. After overnight slow destain, the images were viewed under the white light (UVP, Cambridge, UK).

### Bioinformatics predictions prior to purification of S and F proteins

Bioinformatics tools were used to predict the theoretical iso-electric point (pI) and molecular weight (Mw) of both proteins. The direct strand of the nucleotide sequence for each protein subunit including the six histidine moieties were translated into protein using the nucleotide-protein translation tool (http://www.ebi.ac.uk/Tools/emboss/transeq/index.html) provided by the European Bioinformatics Institute (EBI). To infer the structural bioinformatics image of the two pro-toxins, we entered the sequences obtained from transeq into the protein structure prediction server (PS)2 (http://ps2.life.nctu.edu.tw/) provided online by the National Chiao Tung University Molecular Bioinformatics Centre. PS^2^ is an automated homology modelling server. The method uses an effective consensus strategy by combining PSI-BLAST, IMPALA, and T-Coffee in both template selection and target-template alignment. The final three dimensional structure is built using the modeling package MODELLER [36].

### Preparation of his-tagged pvl protein subunits for chromatographic purification

Following transeq translation, the theoretical pI and Mw of the protein subunits were computed by entering the sequences of single letter protein codes into the pI analysis tool at EXPASY (http://www.expasy.org/tools/pi_tool.html). Preparation of His-tagged PVL protein subunits followed a protocol used in our laboratory for other protein work [34,37], with modifications. Briefly, the bulk of the *E. coli* cells expressing the fusion proteins pelleted and saved at -80°C were gently resuspended in 30 ml cold charge buffer (20 mM Imidazole pH 7.0, 50 mM NaCl, 10% (v/v) glycerol, 1 mM PMSF) and then sonicated on ice for 20 minutes (intermittent 15 seconds bursts of 10 microns) using Soniprep 150 Plus Ultrasonic Disintegrator (MSE, London, UK). Two successive 30 minutes centrifugations at 18000 g were used to clear the cell lysate of insoluble cell debris ready for purification by Nickel affinity (Ni^++^-affinity) chromatography.

### Purification of his-tagged pvl protein subunits by nickel-affinity chromatography

Nickel-affinity chromatography (NAC) was performed using fast protein liquid chromatography (FPLC) system (BioLogic LP: Bio-Rad, USA). A communal protocol used for Ni^++^-affinity FPLC in our laboratory [38], was used for this study, with some modifications. The schema of the FPLC programme is detailed in Table 1. Briefly, a 5 mL HiTrap chelating HP column, Code Number: 17-0409-01 (GE Healthcare, Amersham Biosciences, Buckinghamshire, UK) was charged with Nickel using a single 20 ml injection of 50 mM NiCl_2_, equilibrated in buffer A (25 mM NaH_2_PO_4_ pH 7.0, 0.5 M NaCl, 5% (v/v) glycerol, 2 mM imidazole), then buffer B (25 mM NaH_2_PO_4_ pH 7.0, 0.5 M NaCl, 5% (v/v) glycerol, 1 M imidazole), finally buffer A again. Solid NaCl was added to the cleared cell lysate to a final concentration of 0.5 M and the sample applied to the column at a flow-rate of 1 ml/min. The loaded column was washed in 5% buffer B (15 mL), which is 95% buffer A, until no unbound proteins eluted from the column which is monitored in real-time via the chromatogram generated by the BioLogic LP software. Bound proteins were eluted from the column using a linear gradient of 5-85% buffer B in a total volume of 40 ml. The purity and relative Mw of the fusion proteins were assessed by SDS-PAGE (10%).

### Preparation of rLukS-PV for further purification by ion exchange chromatography

The protein was dialysed overnight in buffer A (10 mM Tris, 50 mM NaCl, pH 7.0) using 8000 pore membrane (Millipore, UK). All buffers and solutions intended to run through the system, including water, were degassed before they getting in contact with the AKTA Explorer system.

### Purification by ion exchange chromatography

Ion exchange chromatography (IEC) was performed using ÄKTA™ Explorer 10 FPLC (Amersham Pharmacia Biotech, USA) driven by UNICORN 4 software. The buffers and protocol are similar to those described elsewhere [38,39]. Briefly, the contents of the tubes bearing the S-subunit were pooled together and loaded into the AKTA Explorer FPLC system. The S-loop was filled with degassed double distilled water and attached to the system. The IEC was programmed to bind protein in low salt buffer A (same buffer A used for NAC) and elute in high salt gradient IEC buffer B (10 mM Tris, 1.0 M NaCl, pH 7.0). Buffer A was the donor of positive charge, while buffer B is the ion exchange gradient buffer. Use of Mono S column for IEC had been described elsewhere [39]. Briefly, following a system wash, the protein was injected into the sample inlet of the ÄKTA™ explorer while buffers A and B were loaded into inlets A11 and B1 respectively. The Mono S column was attached to the nozzle between valves V2 and V3 when V2 and V3 were in position 2. The alarm monitors were set to a limit of 3.2 MPa and enabled. The flow-path for the column position was set to position 2. Degassed double distilled water was allowed to flow through the column at a rate of 1 ml/minute for 5 minutes. Another system wash was performed. Water was replaced with buffer A into inlet A11 and buffer B into inlet B1. The protein in buffer A was loaded into the S-loop. The chromatography was performed by allowing the contents of the S-loop to load unto the column at a rate of 1.0 ml/minute.

### Further Purification of rLukS-PV by size exclusion (gel filtration) chromatography

Except for the few differences in instrumentation mentioned below, the protocol and reagents used for size exclusion chromatography (SEC) and for the IEC were the same. We replaced the Mono S column used for IEC with a HisPrep™ Superdex 30 column previously used in our laboratory for SEC of protein sizes similar to the 6His-LukS-PV [34,37]. HisPrep™ columns are designed for scaling up the purification of His-tagged proteins and have 1/16" fittings for convenient use on the ÄKTA™ Explorer FPLC system.

## Results and discussion

### Recombinant lukS-PV and lukF-PV generated from S. aureus MW2

**Figure 1:**
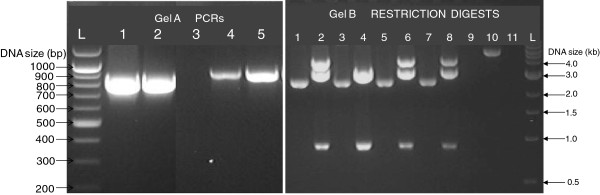
**Start-up amplification and restriction digests of rlukS-PV and rlukF-PV. A.** PCR amplification of *rlukS-PV* and *rlukF-PV* from *S. aureus* MW2 template genomic DNA. Lane L, 100 bp DNA Marker (NEB, UK); Lanes 1 and 2, rlukS-PV, 868 bp; Lane 3, Molecular grade water used as PCR negative control; Lanes 4 and 5 rlukF-PV, 919 bp. **B.** Double digests (*NcoI* and *XhoI*) of minipreps of the intermediate host *E. coli* 5α-pGEMT-Easy-luk-PV to release the insert DNA fragments. Lane 1, Undigested pGEMT-Easy-rlukF-PV; Lane 2, Digested pGEMT-Easy-rlukF-PV showing the cleaved insert rlukF-PV below the 1.0 kb mark; Lane 3, Duplicate of lane 1; Lane 4, Duplicate of lane 2; Lane 5, Undigested pGEMT-Easy-rlukS-PV; Lane 6, Digested pGEMT-Easy-rlukS-PV showing the cleaved insert rlukS-PV, below the 1.0 kb mark; Lane 7, Duplicate of lane 5; Lane 8, Duplicate of lane 6; Lane 9, Undigested pET expression vector; Lane 10, pET vector cut with NcoI and XhoI (the next home of the cleaved insert DNA fragments); Lane 11, Molecular grade water; Lane L, 1 kb DNA Marker (NEB, UK).

Primer sequences used for the amplification of *rlukS-PV* and *rlukF-PV* from the *S. aureus MW2* DNA are presented in Table 1. PCR primers were synthesised by SigmaGenosys (Sigma, UK). DNA fragments (*rlukS-PV* and *rlukF-PV*) amplified from *S. aureus* MW2 are shown (Figure 1A). For easy identification, the *rlukF-PV* fragment is the larger amplicon (Lanes 1 and 2, 919 bp), while the *rlukS-PV* is the smaller amplicon (Lanes 4 and 5, 868 bp), thus easily giving clue of successful and reliable amplification of the two DNA fragments. Amplification errors can be mistaken for polymorphisms or mutations and loss of PCR amplification product or wrong size is not unusual in gene cloning work. We were very careful to use optimally working PCR inputs, including high fidelity proofreading *Pwo* DNA polymerase and fresh template DNA. Importantly too, the design of the oligonucleotide primers was done with much precision. Also, we avoided carryover of DNA from other experiments as sequencing results and blastn showed 100% specificity in the lengths and nucleotide contents of the amplified fragments. The success of the experiments at this primary stage was crucial to the generation of the recombinant DNA for engineering into the intermediate *E. coli* host systems.

### Sub-cloning of insert rlukS-PV and rlukF-PV from intermediate system into expression system confirmed by culture and PCR

**Figure 2:**
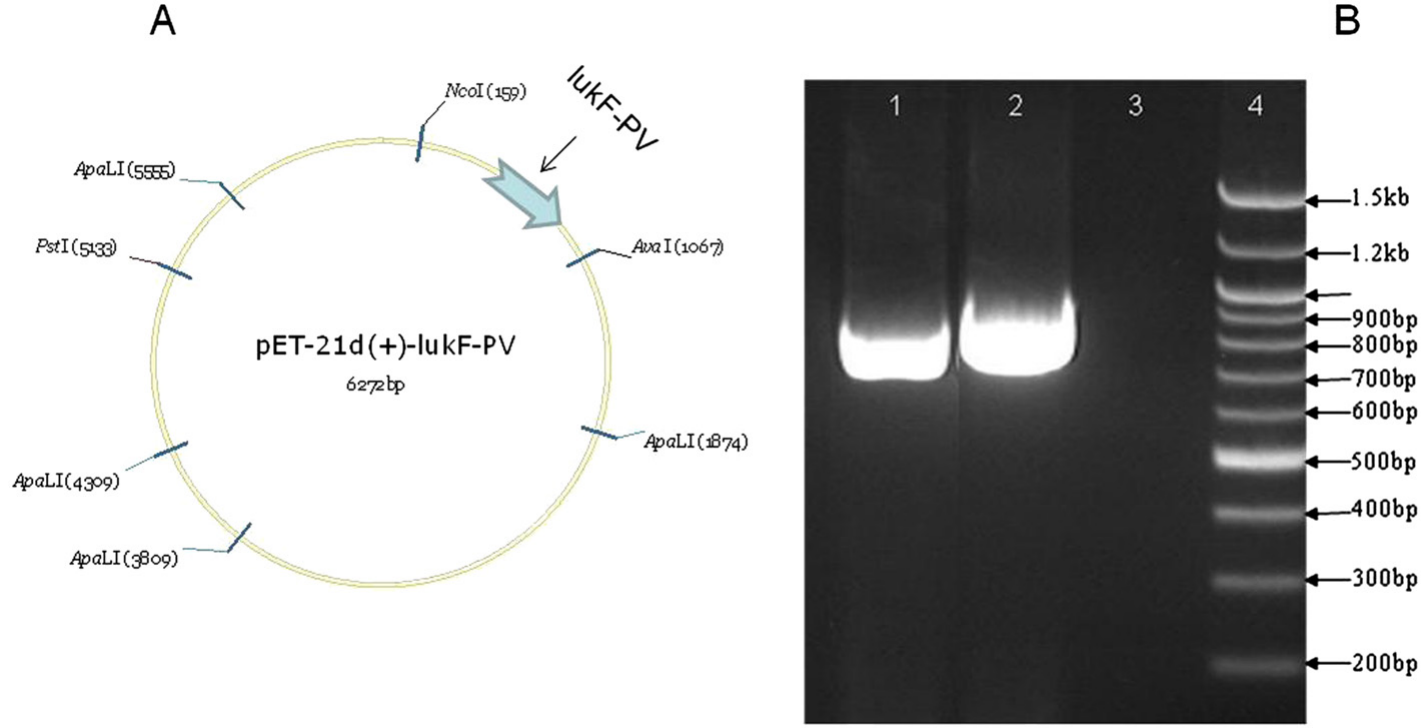
**Reconstruction and amplification of *rlukS-PV *and *rlukF-PV*from protein expression system. A.** Gene map of pET-21d(+)-lukF-PV (Reconstructed using VECTOR NTI software). The insert (*rlukF-PV*) locates within the *NcoI* and *AvaI* RDE sites, technically between the T7 promoter and terminator, all confirmed by sequencing. NOTE: The VECTOR NTI prefers the non-specific nuclease (AvaI), which recognises the degenerate sequence (CYCGRG), over the specific XhoI used in the cloning experiments which specifically recognises the sequence (CTCGAG) engineered into the primers used for recombination. **B.** Amplification of *rlukS-PV* and *rlukF-PV* from the expression system. Lane 1, *rlukS-PV* amplified from expression *E. coli* BL21(DE3)-pET-21d(+)-lukS-PV; Lane 2, *rlukF-PV* amplified from expression *E. coli* BL21(DE3)-pET-21d(+)-lukF-PV; Lane 3, Molecular grade water used as negative PCR control; Lane 4, 100 bp DNA ladder.

The plasmids used for this work are listed in Table 2. Since the pGEM®-T Easy Vector harbours Amp^r^ gene which confers ampicillin resistance, the presence of the insert within the intermediate host system was confirmed by growth on LB agar supplemented with amplicillin (100 μg/mL). Transformants were further confirmed by PCR amplification of each insert from the respective intermediate host system. The amplicons were the same as in Figure1A (no need repeting images). Following the cleaving of the insert DNA fragments of *rlukS*-*PV* and *rlukF*-*PV* off the miniprep of the intermediate host system using *Nco*I and *Xho*I RDEs (Figure 1B), the DNA fragments were ligated with pET-21d(+) which had been restricted with the same *NcoI* and *XhoI*. In principle, a molecule of the DNA insert ligated into a molecule of the restricted and linearized plasmid vector and kept gated by dephophorylation. Following ligation, the insert fragment becomes integrated into the plasmid vector which becomes recircularized as illustrated with the *lukF*-*PV* fragment (Figure 2A). Following the ligation of the plasmids with the insert DNA fragments, the ligation products were separately transformed into *E. coli* BL21(DE3). Recombination was confirmed by the growth of the transformant *E. coli* BL21(DE3)pET-rlukS-PV and *E.coli* BL21(DE3)pET-rlukF-PV on LB agar supplemented with ampicillin (100 μg/mL). Further confirmation was based upon the amplification of the appropriate PCR products from the isolated colonies of the expression *E. coli* hosts (Figure 2B). Proof of transformation has been successful elsewhere by ampicillin agar cultures [38,40] and genomic PCR of the transformant colony for the insert DNA [41].

**Table 2:** Primers designed in this study and used for recombination

**Primer Id**^**a**^	**Primer sequence in 5**′ → **3**′ **direction**^**b**^	**Amplicon**
		**(Size in bp)**
rlukS-PV-1	GG**C/CATGG**ATAACAATATTGAGAATATTGGTGATGG	*rlukS-PV* (868 bp)
rlukS-PV-2	GGC**C/TCGAG**ATTATGTCCTTTCACTTTAATTTCATGAG	
rlukF-PV-1	GGC**C/CATGG**CTCAACATATCACACCTGTAAG	*rlukF-PV* (919 bp)
rlukF-PV-2	GGC**C/TCGAG**GCTCATAGGATTTTTTCCTTAGATTGAG	

^a^ Primer identification.

^b^ RDE sequences bold and underlined.

### Nucleotide sequencing confirms the structural integrity of the reading frames

Nucleotide sequence of the recombinant DNA amplified from the expression *E. coli* genome (Additional file 1 and Additional file 2) and the translated protein products for respective fusion proteins are presented (Additional file 3 and Additional file 4). At this stage of the experimentation, establishment of clones with perfect (100%) identity, were very encouraging for us to rule out mutations which could generate aberrant proteins. Ranging from the stringency of the *Pwo* used for initial amplification of the recombinant DNA, through every step of the cloning, we are pleased with the outcome. The accuracy in size and structure of the sequences are good rewards for all the effort.

### SDS-PAGE shows over-expression and relative sizes of S and F protein subunits

**Figure 3:**
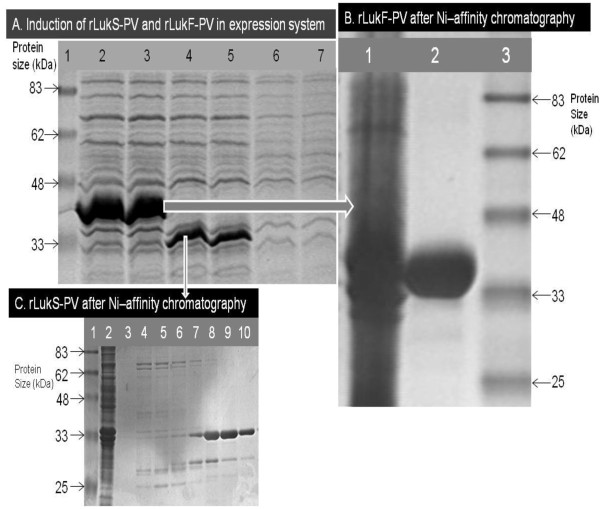
**Expression and Ni**^**++**^**-affinity purification of S and F protein subunits. A.** Over-expression of fusion LukS-PV and LukF-PV (10% SDS-PAGE) Lane 1, Protein Mw standard; Lanes 2 & 3, Total soluble lysate of *E. coli BL21*(DE3)pLysS-pET-21d(+)-lukF-PV after induction with IPTG; Lanes 4 & 5, Total soluble lysate of *E. coli BL21*(DE3)pLysS-pET-21d(+)-lukS-PV after induction with IPTG; Lane 6, Uninduced *E. coli* BL21(DE3)-pET-21d(+)-lukS-PV; Lane 7, Un induced *E. coli* BL21(DE3)-pET-21d(+)-lukF-PV. 3B and C. Purity of His-Tagged LukS-PV and LukF-PV following Nickel Affinity chromatography. **B.** Lane 1, Total soluble lysate of *E. coli BL21*(DE3)pLysS-pET-21d(+)-lukF-PV after induction with IPTG; Lane 2, Purified 6His-LukF-PV; Lane 3, Protein Mw standard. **C.** Purity of His-Tagged LukS-PV following Nickel Affinity chromatography. Lane 1, Protein Mw standard; Lane 2, Total soluble lysate of *E. coli BL21*(DE3)pLysS-pET-21d(+)-lukS-PV after induction with IPTG; Lane 3, water; Lanes 4, 5 & 6, Eluate without His-Tagged LukS-PV; Lanes 7, 8, 9 & 10, Eluate containing His-Tagged LukS-PV.

SDS-PAGE showed clear differences in the purity and relative molecular sizes of the two proteins (Figure 3A). The 6His-LukS-PV located approximately at the 33 kDa mark while the 6His-LukF-PV band was definitely above the 33 kDa mark. This inference corroborates previous reports elsewhere which documented the relative molecular sizes of 34 kDa and 33 kDa for LukF-PV and LukS-PV respectively [3,4]. Interestingly, lanes 2 and 3 of Figure 3A showed very strong expression of the F subunit, while lanes 4 and 5 show expression of the S subunit, though not as strong as F expression. Whereas the concentration of IPTG used and the duration of induction were the same for both proteins, whatever might be responsible for the obvious difference in the expression of the two proteins is yet unknown to us. However, the major essence is that both proteins showed over-expression and availability for purification.

### Protein purification by nickel-affinity chromatography shows dissimilar yields

**Figure 4:**
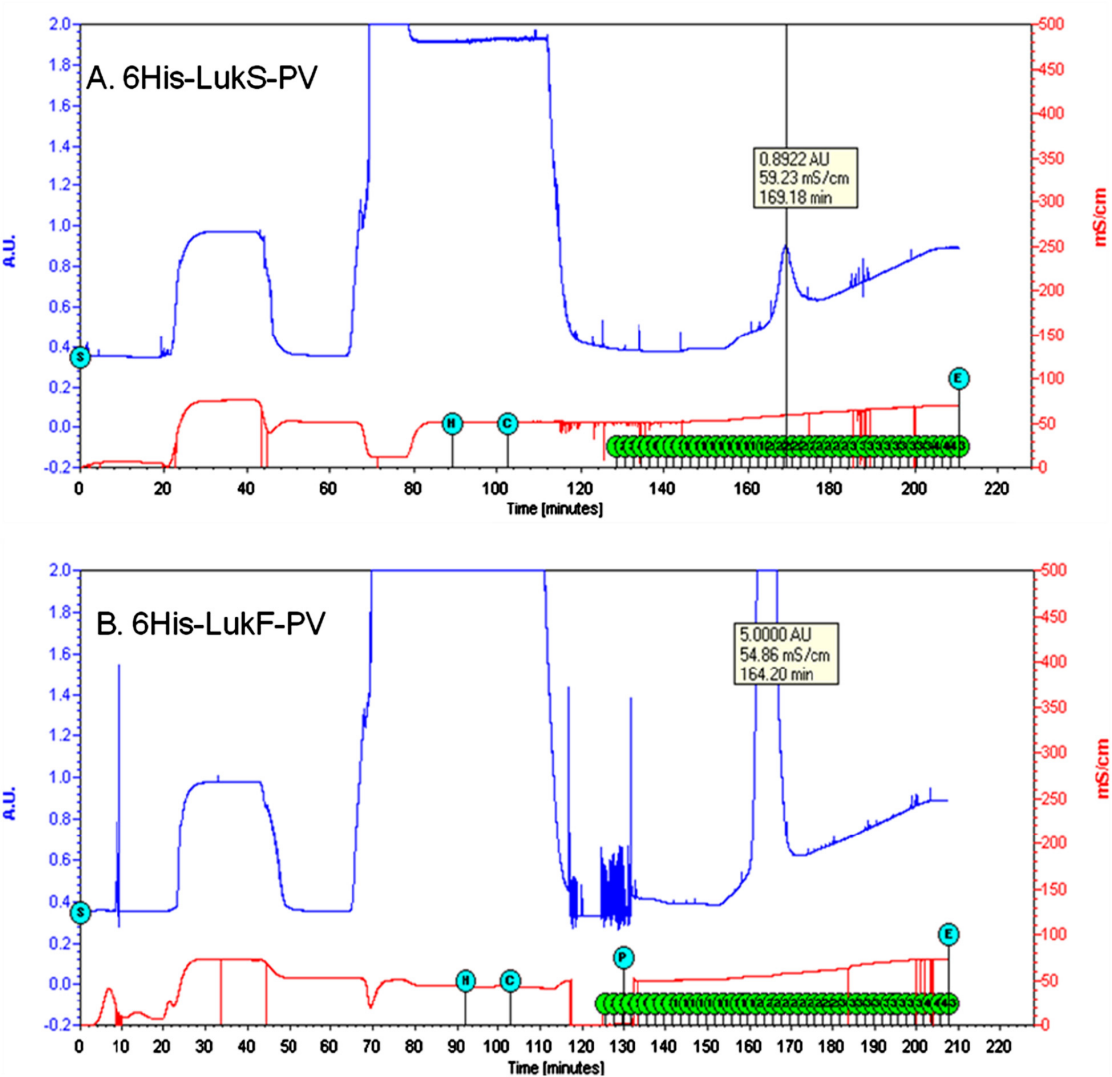
**BioLogic LP chromatogram during Ni**^**++**^**-affinity chromatographic purification of 6His-LukS-PV (upper) and 6His-LukF-PV (lower) in real-time.** NOTE: AU = Absorbance unit, mS/cm = conductance of mobile phase. The inscriptions within the blue circles are the operational mode, i.e., S = start, H = hold, P = Pause, C = continue, and E = end. 6His-LukS-PV purification: The sign-post shows a low concentration of protein (0.8922 AU) in the eluate. **B** 6His-LukF-PV purification: The sign-post shows a high concentration of protein (5.000 AU) in the eluate.

The protocol for Ni^++^-affinity chromatography is summarized schematically in Table 3. The SDS-PAGE of lysate and eluate following Ni^++^-NTA chromatography of 6His-LukS-PV and 6His-LukF-PV are shown (Figure 3B and C). The F subunit came out of the Ni-affinity chromatography with no band of impurity (Figure 3B), while the S subunit had bands of impurity above and below the expected protein band. We do not know if the expression patterns of the proteins contributed to the variable purification efficiency of NAC which is obvious in Figure 3. Similar reduced efficiency of purification of LukS-PV was reported following a work in which both the S and F subunits were purified from lysates of *S. aureus* USA300 and USA400; they had to perform a second round of ion exchange chromatography. Ours is the second report of such reduced purification efficiency. It is possible that some yet unidentified amino acid moieties within the sequence of the S protein have the capacity to reduce the efficiency of purification of the S protein by currently known technology. Though that was not the theme of our experiments, but in observing that it has happened to other researchers before us, we would like to suggest that future attempt at resolving such issues might be helpful to the scientific community. On the chromatogram generated by the BioLogic LP software during the purification (Figure 4), the value of the absorbance unit (AU), graduated in blue colour on the left hand side bar of the vertical axis of the chromatographic purification plot, which stood at 0.8922 showed that the concentration of His-tagged protein eluting from the system was low (Figure 4A). The purification plot for rLukF-PV showed that the AU was at the maximum mark of 5.0000 (Figure 4B) which is the highest possible output by the BioLogic LP software, an evidence that the F subunit eluted with reckonably high concentration of His-tagged protein. The difference might have to do with the obvious difference in the over-expression of the two proteins (Figure 3A). Having treated the two proteins to the same buffers and protocol, we were unable to explain why the F subunit came off the Ni^++^-affinity chromatography requiring no further purification protocol as no band of impurity was seen on the SDS-PAGE (Figure 3B), whereas the S subunit had several bands of impurity (Figure 3C). Interestingly, a recent report showed that the S subunit purified by ion-exchange chromatography required a second round of ion-exchange chromatography [42].

**Table 3:** **Schema of the Ni**^++^-**NTA fast protein liquid chromatography programme**^**a**,**b**^

**Step**	**Volume (mL)**^**c**^	**Solution**^**d**^
01	00 - 20	D^e^
02	20 - 40	C^f^
03	40 - 60	A
04	60 – 90	E = Protein
05	90 – 115	A
06	115 – 130	Mix 5% B
→ 120 Collect 2 mL		
07	130 - 180	Gradient of 5 to 100% B
08	180 - 200	B
→ Divert to waste		
End of method, washing up of the machine, and log off		

→ No necessary information. Proceed to the shown step.

^a^ The flow-rate was 1.0 mL throughout the entire run.

^b^ The entire programme was 200 mL.

^c^ Increase on the tabulated volumes were always allowed by pushing the Hold (H) button on the machine. This accounts for the ‘H’ on the BioLogic LP output during the run.

^d^ All solutions were filtered and degassed.

^e^ Solution D is sterile distilled water.

^f^ Solution C is the charge buffer.

### Ion exchange chromatography removes some impurity from fusion LukS-PV

**Figure 5:**
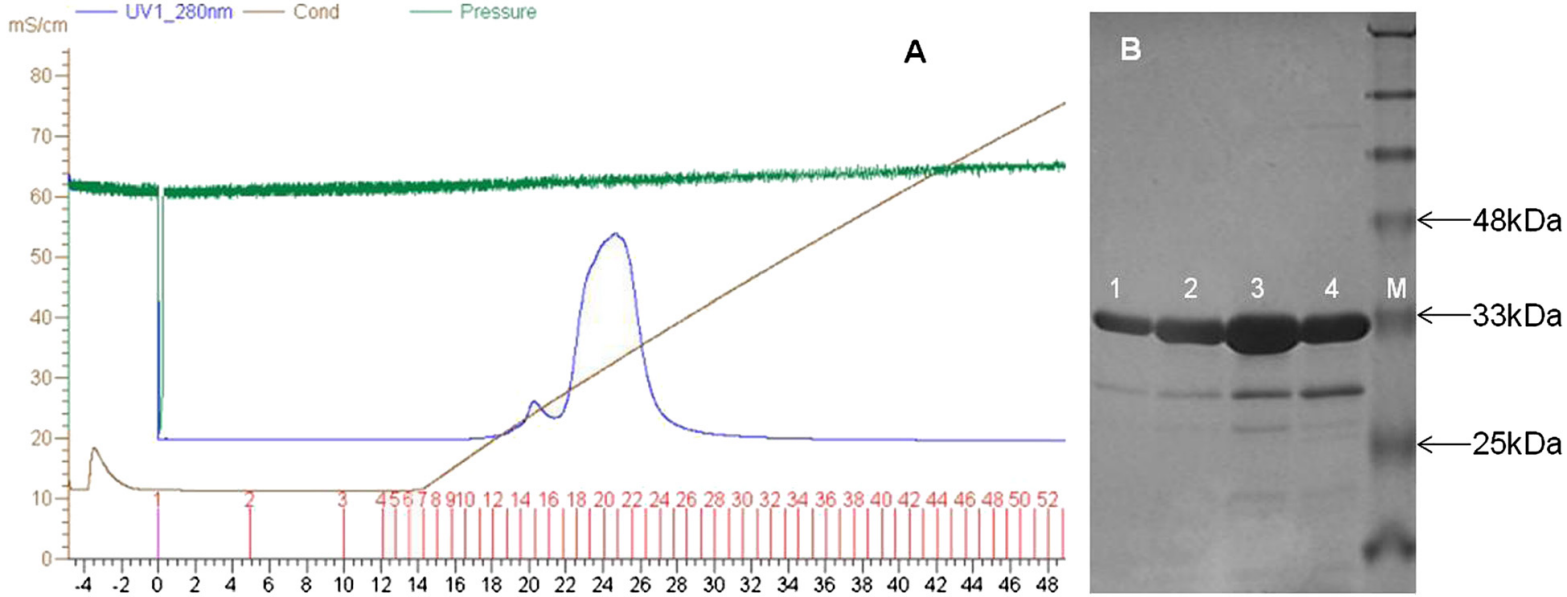
**Ion Exchange Chromatography (IEC) of 6His-LukS-PV. A** AKTA Explorer chromatogram during ion exchange purification of 6His-LukS-PV. NOTE: The blue curve is the entire chromatographic plot itself. On the vertical axis, mS/cm is the conductivity or absorbance, while the green signal is the pressure. On the horizontal axis, the numbers in red (upper) are the identification of the tubes containing the protein, while the grey numbers (lower) is the timer (minutes). **B** Purity of 6His-LukS-PV following purification by IEC. Lanes 1, 2, 3 & 4, All of the eluate had bands of impurity between the 33 kDa and 25 kDa Mw marks. Lane M, Protein Mw standard.

The characteristics of the Mono S column used for IEC are described in Table 4. Those characteristics were compatible with the ÄKTA™ Explorer 10 FPLC system. The purification plot during ion exchange chromatography and the SDS-PAGE of rLukS-PV following the ion exchange are shown (Figure 5). The SDS-PAGE of the eluate from IEC showed that there was no band of impurity above the 33 kDa band. However, the bands of impurity below the 33 kDa band of the S subunit were not removed by IEC. Obviously, further purification treatment is required. Graves and colleagues found that a second round of ion exchange chromatography was necessary after the first attempt [42].

**Table 4:** **Characteristics of mono S column used for ion exchange chromatography**^**a**^

**Property**	**Description**
Dimensions	50 × 5 mm
Volume	1 mL
Flow rate	0.5-2 mL/min
Max. backpressure	750 psi (5 MPa)
Temperature	4°-40°C
pH	2-12
Approximate separation time	5 minutes

^a^The manufacturers manual supports that Mono S column is compatible with AKTA systems.

### Size exclusion chromatography purified fusion LukS-PV ultimately

**Figure 6:**
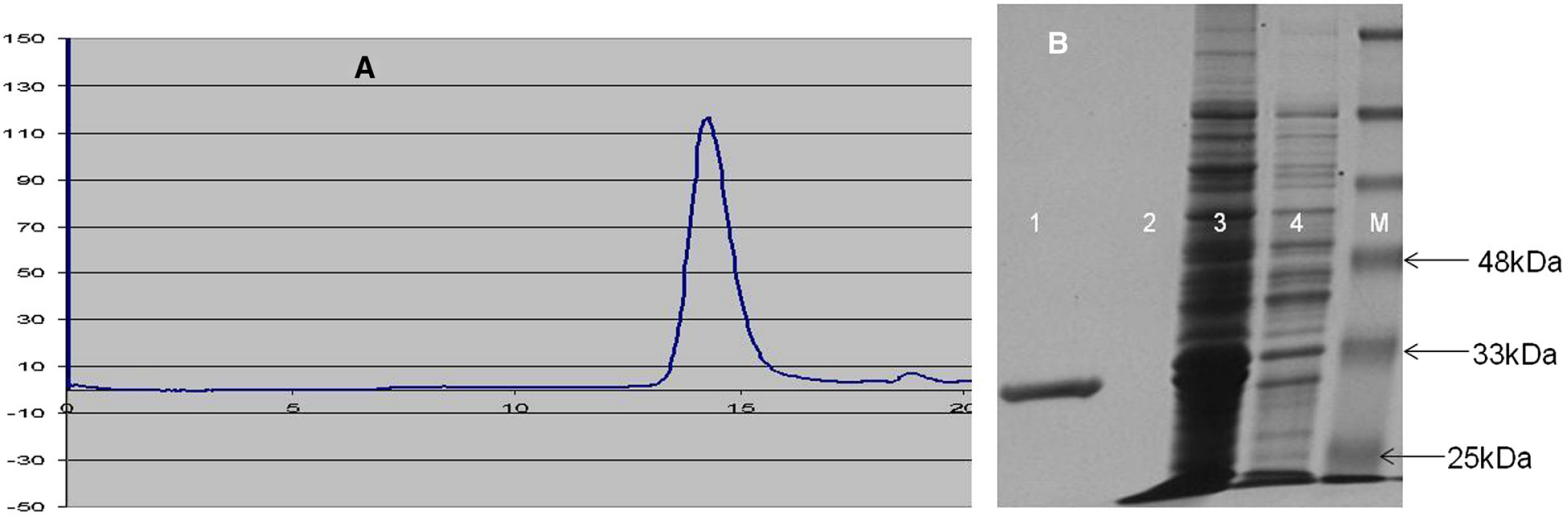
**Size Exclusion Chromatography (SEC) of 6His-LukS-PV. A** AKTA Explorer Chromatographic plot during SEC purification of 6His-LukS-PV. NOTE: Vertical axis = absorbance, Horrizontal axis = Time (minutes). **B** Purity of 6His-LukS-PV following purification by SEC. Lane 1, 6His-LukS-PV completely pure without bands of impurity; Lane 2, water; Lane 3, Total soluble lysate of *E. coli BL21*(DE3)pLysS-pET-21d(+)-lukS-PV after induction with IPTG; Lane 4, *E. coli BL21*(DE3)pLysS-pET-21d(+)-lukS-PV before induction with IPTG, Lane M, Protein Mw standard.

Running the same buffers as for the IEC with replacement of Mono S column by HisPrep™ Superdex 30 column, removed the rest of the impurities leaving the S subunit ultra pure as bands of impurity were not seen on the SDS-PAGE, neither above nor below the S subunit mark (Figure 6). Size exclusion is a well known method of purifying proteins according to molecular sizes. Exploiting the interactions between poly-histidine fusion tags and immobilized metal ions has been well documented [39]. Like the 6His-LukF-PV subunit reported here, many proteins have been purified successfully by single-step Nickel affinity chromatography, including UreE [43]. Some proteins, however, tend to elute with impurities after one chromatographic treatment. LukS-PV from USA300 and USA400 did not become pure until after a second round of ion exchange chromatography [42].

### Molecular weight (Mw) of peptides determined by concordance between bioinformatics prediction, SDS-PAGE and mass spectrometry

**Figure 7:**
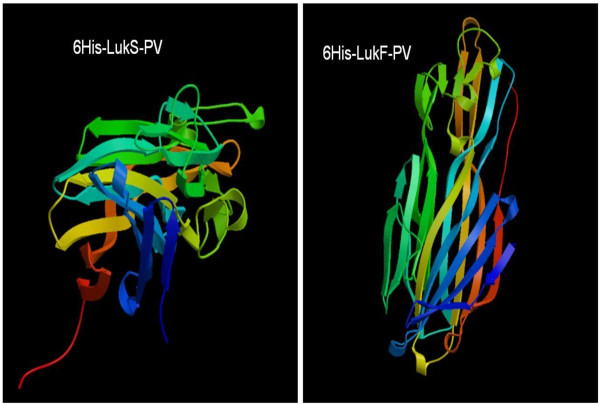
**Structures of 6His-LukSPV and 6His-LukF-PV as predicted by Protein Structure Prediction Server (PS)2 (http://ps2.life.nctu.edu.tw/).**

The translated protein sequences for rLukS-PV and for rLukF-PV are presented in the additional file at the end of the document (Additional file 1). The EXPASY predicted Mw for the F peptide was 35645.41 Da and for the S peptide was 33530.04 Da respectively. All the SDS-PAGE gels were in concordance with the EXPASY prediction as the F peptide was always far above the 33 kDa mark while the S peptide was always close to the 33k Da mark in all the SDS gels used in this work (Figures 3, 5 and 6). Results of the structure prediction for the translated protein sequences are presented (Figure 7). The mass spectrometry deconvolution data showed Mw of 33528.34 Da and 35643.57 Da for the S and F subunits respectively. To all intents and purposes, the concordance between the EXPASY prediction, the SDS-PAGE and the mass determination by MS suggests that all three tools could be relied upon to predict, estimate or measure the Mw of PVL subunits as long as the user understands the strengths and limitations of each of the tools.

## Conclusions

Our aim to provide biological tools for possible translation into anti-PVL therapy was driven by expert opinion on the menacing PVL syndrome. As the risk level of PVL-positive *S. aureus* is yet to be ascertained, manipulations of the *S. aureus* substrain MW2 (USA400) from NARSA were carried out in the BSL3. Given the several life-threatening conditions associated with USA400 especially, we would rather apply higher safety precautions than actually required to guarantee the safety of our laboratory users. This step was very important to us and the outcome was quite rewarding as there has been no record of our laboratory users/visitors, both the BSL3 and the BSL2, including the adjacent offices, coming down with PVL-syndrome, even long after completion of our experiments.

We are happy with the outcome of the DNA sequencing and BLAST which we used to confirm molecular identity at all necessary stages. The pET-21d(+) vector was chosen because it carries an N-terminal T7•Tag® sequence and a C-terminal 6His•Tag® sequence. The T7 and 6His-Tag motifs are very important for expression and for down-stream treatments including purification by Nickel-affinity chromatography. Therefore, it was necessary to send the DNA out of our facility to be sequenced by the Cambridge Geneservice as they had the T7 terminator primers which were not available in Nottingham at the time.

The reliability of the BioLogic-LP and AKTA systems to guide the progress of the purification cycle in real-time is commendable. The purification plots showed that the absorbance at any point during the elution correlated directly with the concentration of His-tagged protein in the eluate (Figures 4, 5 and 6). Also, the SDS-PAGE used to infer protein purity and molecular size showed that the relative intensity of the protein bands eluted from the system correlates with the AU observed during the run. This is one of the innovative and user-friendly features of modern chromatographic systems. The fact that the experimenter can readily identify the tube(s) containing the His-tagged proteins is very helpful as it reduces waste of time and materials. Though the initial cost of purchasing any of the modern chromatographic instruments might be perceived as high, in the end they are cost-effective.

Taken together, we engineered the *lukS*-*PV* and *lukF*-*PV* genes respectively encoding the S and F subunits of *S. aureus* PVL toxin from *S. aureus* stain USA400 into separate *E. coli* expression host systems. Having purified the pro-toxin subunits, we supplied them to the Nottingham Cancer Immunotherapy group who used them to establish anti-PVL monoclonal antibodies. We are happy that the pro-toxin subunits have served as useful input for the establishment of sequence-based diagnostic and therapeutic tools for rapid and specific diagnosis and abrogation of PVL-associated leukocytolysis and the attendant rapid deterioration of its victims. In keeping with the University of Nottingham policies on genetically modified organisms, we handed over the engineered *E. coli* clones expressing the PVL pro-toxin subunits, namely *E. coli BL21*(*DE3*)-*pET*-*21d*(+)-*lukS*-*PV* and *E. coli BL21*(*DE3*)-*pET*-*21d*(+)-*lukF*-*PV*, for LukS-PV and LukF-PV respectively, to the Biosafety Officer (AC) for safe-keeping.

## Competing interests

The authors declare that they have no competing interests.

## Authors’ contributions

CEO conception and design, all the experimentation, analysis and interpretation of data, drafting and revising the manuscript. AC design and oversight of work in BSL3 workspace, instrumentation, biosafety and biocontrol, supervision, analysis and interpretation of data, and revising the manuscript. CP supervision, analysis and interpretation of data, and revising the manuscript. RJ acquisition of funding, conception, analysis and interpretation of data, general supervision of the research group, networking the supplies of purified toxin subunits, and revising the manuscript. All authors read and approved the final manuscript.

## Supplementary Material

Additional file 1 **Direct strand sequence of *rlukS-PV *with 3ʹ terminal 6-CAC tag as present in the expression system.** Click here for file

Additional file 2 **Direct strand sequence of *rlukF-PV *with 3ʹ terminal 6-CAC tag as present in the expression system.** Click here for file

Additional file 3 **Peptide product (fusion LukS-PV), translated from *rlukS-PV*, with C-terminal 6-Histidine tag as present in the expression system.** Click here for file

Additional file 4 **Peptide product (fusion LukF-PV), translated from *rlukF-PV*, with C-terminal 6-Histidine tag as present in the expression system.** Click here for file
