# Supplementary material for: Engineering of the LukS-PV and LukF-PV subunits of Staphylococcus aureus Panton-Valentine leukocidin for Diagnostic and Therapeutic Applications
Source: BMC Biotechnol. 2013 Nov 19;13:103. doi: 10.1186/1472-6750-13-103 (PMC3870988; doi:10.1186/1472-6750-13-103)
Supplement: Additional file 1 — Direct strand sequence of rlukS-PV with 3ʹ terminal 6-CAC tag as present in the expression system. [file 1472-6750-13-103-S1.doc]

**Additional Files**

**Appendix 1.** Direct strand sequence of *rlukS-PV* with 3ʹ terminal 6-CAC tag as present in the expression system

...ATGGATAACAATATTGAGAATATTGGTGATGGCGCTGAGGTAGTCAAAAGAACAGAAGATACAAGTAGCGATAAGTGGGG

GGTCACACAAAATATTCAGTTTGATTTTGTTAAAGATAAAAAGTATAACAAAGACGCTTTGATTTTAAAAATGCAAGGTT

TTATCAATTCAAAGACTACTTATTACAATTACAAAAACACAGATCATATAAAAGCAATGAGGTGGCCTTTCCAATACAAT

ATTGGTCTCAAAACAAATGACCCCAATGTAGATTTAATAAATTATCTACCTAAAAATAAAATAGATTCAGTAAATGTTAG

TCAAACATTAGGTTATAACATAGGTGGTAATTTTAATAGTGGTCCATCAACAGGAGGTAATGGTTCATTTAATTATTCAA

AAACAATTAGTTATAATCAACAAAACTATATCAGTGAAGTAGAACGTCAAAATTCAAAAAGTGTTCAATGGGGAATAAAA

GCTAATTCATTTATCACATCATTAGGTAAAATGTCTGGACATGATCCAAATTTATTTGTTGGATATAAACCATATAGTCA

AAATCCGAGAGACTATTTTGTTCCAGACAATGAATTACCCCCATTAGTACACAGTGGTTTCAATCCTTCATTTATTGCAA

CTGTTTCTCATGAAAAAGGCTCAGGAGATACAAGTGAATTTGAAATAACGTATGGCAGAAATATGGATGTTACTCATGCT

ACTAGAAGAACAACACACTATGGCAATAGTTATTTAGAAGGATCTAGAATACACAACGCATTTGTAAACAGAAATTACAC

AGTTAAATATGAAGTGAACTGGAAAACTCATGAAATTAAAGTGAAAGGACATAATCTCGAG**CACCACCACCACCACCAC...**
