# Supplementary material for: Engineering of the LukS-PV and LukF-PV subunits of Staphylococcus aureus Panton-Valentine leukocidin for Diagnostic and Therapeutic Applications
Source: BMC Biotechnol. 2013 Nov 19;13:103. doi: 10.1186/1472-6750-13-103 (PMC3870988; doi:10.1186/1472-6750-13-103)
Supplement: Additional file 2 — Direct strand sequence of rlukF-PV with 3ʹ terminal 6-CAC tag as present in the expression system. [file 1472-6750-13-103-S2.doc]

Additional file

### Appendix 2. **Peptide product** (fusion LukS-PV), **translated from** *rlukS-PV*, with C-terminal 6-Histidine tag as present in **the** expression system

MDNNIENIGDGAEVVKRTEDTSSDKWGVTQNIQFDFVKDKKYNKDALILKMQGFINSKTT

YYNYKNTDHIKAMRWPFQYNIGLKTNDPNVDLINYLPKNKIDSVNVSQTLGYNIGGNFNS

GPSTGGNGSFNYSKTISYNQQNYISEVERQNSKSVQWGIKANSFITSLGKMSGHDPNLFV

GYKPYSQNPRDYFVPDNELPPLVHSGFNPSFIATVSHEKGSGDTSEFEITYGRNMDVTHA

TRRTTHYGNSYLEGSRIHNAFVNRNYTVKYEVNWKTHEIKVKGHNLEHHHHHH
