# Supplementary material for: Engineering of the LukS-PV and LukF-PV subunits of Staphylococcus aureus Panton-Valentine leukocidin for Diagnostic and Therapeutic Applications
Source: BMC Biotechnol. 2013 Nov 19;13:103. doi: 10.1186/1472-6750-13-103 (PMC3870988; doi:10.1186/1472-6750-13-103)
Supplement: Additional file 3 — Peptide product (fusion LukS-PV), translated from rlukS-PV, with C-terminal 6-Histidine tag as present in the expression system. [file 1472-6750-13-103-S3.doc]

Additional file

### Appendix 3. **Direct strand sequence of *rlukF-PV* with** 3ʹ **terminal** 6-CAC **tag as present in the expression system**

...ATGGCTCAACATATCACACCTGTAAGTGAGAAAAAGGTTGATGATAAAATTACTTTGTACAAAACAACTGCAACATCA

GATTCCGATAAGTTAAAAATTTCTCAGATTTTAACTTTTAATTTTATTAAAGATAAAAGTTATGATAAAGATACATTAAT

ACTCAAAGCTGCTGGAAACATTTATTCTGGCTATACAAAGCCAAATCCAAAAGACACTATTAGTTCTCAATTTTATTGGG

GTTCTAAGTACAACATTTCAATTAATTCAGATTCTAATGACTCAGTAAACGTTGTAGATTATGCACCTAAAAATCAAAAT

GAAGAATTTCAAGTACAACAAACGGTAGGTTATTCTTATGGTGGAGATATTAATATCTCTAACGGCTTATCAGGTGGAGG

TAATGGTTCAAAATCTTTTTCAGAGACAATTAACTATAAACAAGAAAGCTATAGAACTAGCTTAGATAAAAGAACTAATT

TCAAAAAAATTGGTTGGGATGTTGAAGCACATAAAATTATGAATAATGGTTGGGGACCATATGGCAGAGATAGTTATCAT

TCAACTTATGGTAATGAAATGTTTTTAGGCTCAAGACAAAGCAACTTAAATGCTGGACAAAACTTCTTGGAATATCACAA

AATGCCAGTGTTATCCAGAGGTAACTTCAATCCAGAATTTATTGGTGTCCTATCTCGAAAACAAAACGCTGCAAAAAAAT

CAAAAATTACTGTTACTTATCAAAGAGAAATGGATAGATATACAAACTTTTGGAATCAACTTCACTGGATAGGTAATAAT

TGTAAAGATGAAAATAGAGCAACTCATACATCAATTTATGAAGTTGATTGGGAAAATCATACAGTTAAATTAATAGATAC

TCAATCTAAGGAAAAAAATCCTATGAGCCTCGAG**CACCACCACCACCACCAC**...
