# Supplementary material for: Engineering of the LukS-PV and LukF-PV subunits of Staphylococcus aureus Panton-Valentine leukocidin for Diagnostic and Therapeutic Applications
Source: BMC Biotechnol. 2013 Nov 19;13:103. doi: 10.1186/1472-6750-13-103 (PMC3870988; doi:10.1186/1472-6750-13-103)
Supplement: Additional file 4 — Peptide product (fusion LukF-PV), translated from rlukF-PV, with C-terminal 6-Histidine tag as present in the expression system. [file 1472-6750-13-103-S4.doc]

### Additional file

### Appendix 4. **Peptide product** (fusion LukF-PV), **translated from** *rlukF-PV*, with C-terminal 6-Histidine tag as present in **the** expression system

MAQHITPVSEKKVDDKITLYKTTATSDSDKLKISQILTFNFIKDKSYDKDTLILKAAGNI

YSGYTKPNPKDTISSQFYWGSKYNISINSDSNDSVNVVDYAPKNQNEEFQVQQTVGYSYG

GDINISNGLSGGGNGSKSFSETINYKQESYRTSLDKRTNFKKIGWDVEAHKIMNNGWGPY

GRDSYHSTYGNEMFLGSRQSNLNAGQNFLEYHKMPVLSRGNFNPEFIGVLSRKQNAAKKS

KITVTYQREMDRYTNFWNQLHWIGNNCKDENRATHTSIYEVDWENHTVKLIDTQSKEKNP

MSLEHHHHHH
